# Novel Plant and fungi-based Alternatives Support Nutritional Adequacy of Diets and Reduce Their Environmental Impacts

**DOI:** 10.1016/j.cdnut.2026.107669

**Published:** 2026-03-12

**Authors:** Sarah Nájera Espinosa, Arli G Zarate-Ortiz, Genevieve Hadida, Jacqueline Tereza da Silva, Alexander Vonderschmidt, Edith Monica Esievo, Tony Carr, Anouk Reuzé, Pauline Scheelbeek

**Affiliations:** 1 Department of Population Health, London School of Hygiene and Tropical Medicine, London, United Kingdom; 2 Centre on Climate Change and Planetary Health, London School of Hygiene and Tropical Medicine, London, United Kingdom; 3 Division of Agriculture and Food Systems, University of Edinburgh, Edinburgh, United Kingdom

**Keywords:** healthy diets, sustainable diets, plant-based alternatives, dietary change, food systems, environment sustainability

## Abstract

**Background:**

Evidence suggests dietary shifts away from animal-sourced toward plant-based foods benefit health and the environment. Although the United Kingdom’s (UK) meat consumption has decreased, further efforts are required to meet national targets. Novel plant- and fungi-based foods (NPBFs) could offer a straightforward alternative to animal-sourced foods with a similar sensory experience. However, their impact on nutrition, the environment, and cost remains unclear.

**Objectives:**

We aimed to identify trade-offs and co-benefits of substituting specific animal-sourced foods with NPBFs in a UK minimally accepted “basic basket” (baseline) stratified by gender.

**Methods:**

Nine new baskets were created to assess the nutritional, environmental, and cost outcomes by substituting either all processed meats, milk, or yogurt with NPBFs (most popular, nutritionally balanced, inexpensive). Foods from the “basic basket” were matched with the UK food composition table. NPBF nutritional data were collected from UK supermarkets using Open Food Facts. All foods were matched with dietary environmental footprints (greenhouse gas emissions, water, and land use). Prices were manually collected from UK supermarkets.

**Results:**

Dietary environmental footprints decreased in all new baskets (‒0.39% to ‒6.94%). Nutritional outcomes varied across new baskets, with most substitutions aligned with recommended weekly averages. Baskets with the most nutritionally balanced NPBFs showed minimal nutritional differences compared to the baseline. Notable micronutrient reductions occurred when replacing milk with the most inexpensive alternative. Nearly all dairy alternative substitutions lowered food basket costs, except for the most popular plant-based drink. All plant-based meats increased the cost.

**Conclusions:**

When the nutritional quality of individual NPBFs is considered, single-targeted replacements of specific animal-sourced foods by NPBFs present a powerful opportunity for health and environmental benefits, especially over processed meats. Without policies to improve NPBFs’ affordability, such a shift at the population level is unlikely, missing an opportunity to drive progress toward net zero and health targets.

## Introduction

Our global food system is negatively affecting the health of our people and planet. Shifting diets in high-income countries by reducing the consumption of animal-sourced foods, especially red and processed meat and dairy, can improve health and environmental outcomes, and animal welfare concerns. The United Kingdom (UK) has seen a pattern of dietary shifts over the last decade, with the consumption of red and processed meat decreasing by ∼17% in the period 2008‒2009 to 2018‒2019 [1]. Despite this reduction, it might be challenging to meet targets as set out by (among others) the National Food Strategy [2] or the Climate Change Committee, with the latter recommending a population-wide meat and dairy reduction of 20‒25% by 2030 and red meat reduction of 35‒40% by 2050 [[3], [4], [5]]. Although much of the environmental discourse has centered on red and processed meat, the environmental impacts of white meat and dairy consumption have received relatively less attention. Although the production of white meat typically results in lower greenhouse gas emissions per 100 g of product than red meat, it is still associated with significant environmental harms, including water, air, and soil pollution, poor animal welfare, and the spread of antimicrobial resistance [6]. Unlike red meat, white meat consumption has increased by 3% in the last decade [1], and is currently the largest contributor to daily meat consumption (42% of chicken and turkey) [7]. Dairy products, particularly cheese, also contribute notably to environmental footprints. However, they are rarely targeted in dietary interventions, partly due to the mixed associations with health, which may be either beneficial [8] or neutral [9]. Reducing meat consumption remains a key priority for 2 main reasons: first, it offers greater potential for lowering environmental impacts compared to reductions in dairy intake [10]. Second, processed meat accounts for almost a third of UK meat consumption (29%), raising concerns given strong evidence linking overconsumption of these foods to increased chronic disease risk [11,12].

There is growing evidence supporting the health and environmental benefits of diets rich in plant-based wholefood such as fruits, vegetables, legumes, tofu, nuts, and whole grains [[13], [14], [15]]. Nevertheless, plant-based wholefoods consumption remains low in the UK [16,17], especially among lower-income households [18]. Despite ongoing efforts by academia, government, and various other key stakeholders to promote healthier and environmentally sustainable eating habits, more substantial and faster dietary shifts are needed—especially given the urgency of the climate crisis.

Novel plant- and fungi-based foods (NPBFs) could potentially contribute to an acceleration of this transition. Designed to mimic the taste and texture of animal-sourced foods, NPBFs aim to reduce the behavioral barriers (cooking knowledge, time, social stigma, and familiarity) often associated with replacing animal-sourced foods with plant-based whole foods [[19], [20], [21], [22]]. Although the market for NPBFs continues to evolve, with demand currently higher among females and younger people, their potential role for healthy and environmentally sustainable food systems has sparked debates across multiple disciplines [[23], [24], [25]].

Due to their novelty, key uncertainties remain regarding their level of processing, methods of production, nutritional composition, health impacts, affordability—particularly for lower-income households—and the broader role NPBFs could play within healthy and sustainable diets. NPBFs are generally classified as ultraprocessed foods due to their manufacturing process and use of food additives. This may contribute to the perception that NPBFs are inherently unhealthy and discourage their consumption [26]. Ultraprocessed foods are designed to replace whole foods and are generally high in energy density, saturated fats, sugar, salt, contain food additives, and low in fiber [27]. However, many NPBFs do not conform to this nutritional profile and, in fact, several individual types and brands of NPBFs align with healthy dietary recommendations [28].

In research where NPBFs have been examined, studies have found that NPBFs are generally healthier and more sustainable than their animal-based counterparts, but these studies usually take 2 main approaches: *1*) product-level comparisons primarily between animal-sourced foods and NPBFs (often across all available products in supermarkets, online retailers, or food composition databases) [[28], [29], [30], [31], [32], [33]], with less work comparing all 3 (animal-sourced foods, plant-based whole foods, and NPBFs) [34]. These studies often lack a broader dietary context, and comparisons for meat usually combine processed and unprocessed foods. Or *2*) dietary modeling studies that assume complete replacement of all animal-based foods [[35], [36], [37]]. Fewer studies have modeled [16,[38], [39], [40]] or conducted trials [41] with partial, selective substitutions of NPBFs within mixed diets.

Although these studies provide valuable insights into partial NPBFs dietary transitions, there is limited research on the role of the most consumed NPBFs in the UK as part of a realistic, socially acceptable and affordable diets that include moderate amounts of animal-sourced foods (e.g., either some lean cuts of meat, fish or some dairy), as opposed to a 100% substitute for animal-sourced foods. This distinction is important because evidence suggests that most consumers of NPBFs often continue to purchase animal-sourced foods rather than adopting a fully plant-based diet [42,43].

In light of the ongoing discussions surrounding plant-based foods and the role of NPBFs in current dietary transitions, this study aims to identify the potential nutritional, economic, and environmental trade-offs and co-benefits of substituting animal-sourced foods with the most commonly consumed NPBFs available to UK consumers. Specifically, we identify the most nutritionally balanced, most inexpensive, and popular NPBF options, based on previous analysis of real take home purchase data of a UK-wide household panel. With cost often an important factor of food choice, particularly among disadvantage communities in the UK [44,45], we applied these substitutions to a predefined minimally acceptable “basic basket” aligning with a realistic, nutritious, and affordable UK food basket that allows individuals to participate in everyday activities accounting for both essential health-related expenditure and the costs of social inclusion [46,47].

With 99% of the population failing to meet national food-based dietary recommendations in the UK [17], and with sociodemographic inequalities in dietary adherence persisting over decades [48], applying national guidelines as a reference diet is impractical. Instead, the “basic basket” was chosen as a more representative benchmark, offering a realistic pathway to improved nutritional adequacy and affordability. By doing so, this study aims to address existing research gaps on the role of the most consume NPBFs in healthy and sustainable diets, and whether NPBFs are economically accessible for society-wide transition by offering evidence-based insights into the most frequently purchased NPBFs divided into 3 groups: *1*) most popular NPBFs; *2*) most nutritionally balanced NPBFs; and *3*) most inexpensive NPBFs. Our findings support a more informed approach to dietary change by offering practical, sustainable, and health-conscious strategies to reduce environmental footprints without compromising taste, affordability, or nutritional quality of diets.

## Methods

### Minimally accepted “basic basket” (baseline) and new baskets

We used the Food Foundation minimally accepted “basic basket” (baseline) for a typical UK diet for a working female (41-y-old female, 70.2 kg, 1.62 m) and man (41-y-old male, 83.6 kg, 1.75 m), as the baseline of our study [46]. The “basic basket” includes a bundle of items (66 items for females and 69 for males) commonly consumed by people from lower-income households in everyday social activities, seasonal events (e.g., Christmas), as well as eating out occasionally. Briefly, the “basic basket” includes items such as minimally processed cereals and grains (pasta, porridge oats, and bread), dairy, meat, fish, eggs, fruits and vegetables, but also incorporates commonly consumed items like snacks (biscuits, crisps), ready meals, coffee, tea, seasonings, and dressings to reflect realistic dietary patterns. The nutritional composition and price of the original “basic basket” are based on a single retailer: Tesco. See Table 1 for a summarized list of the main items included in the basket for males and females. The basket is estimated based on the minimum income standard research based on the collective agreement of what is a “socially acceptable” diet (see O'Connell et al., 2018 [47] and the Food Foundation basic basket tracker [46] for further details). Although the “basic basket” is not a reference diet, this basket was selected as it provides a realistic, socially accepted, reasonably costed, and nutritionally adequate baseline for examining whether wider societal dietary shifts could benefit from replacing certain animal-sourced foods with NPBFs, particularly for individuals from lower socioeconomic backgrounds. See supplementary 2: Table S2.1 and S2.2 for detailed nutritional composition (based on the UK food composition), environmental outcomes, and cost of the basic basket for males and females on a 100 g basis.

**TABLE 1:** Weekly food item list for Females’ and Males’ “Basic Basket” (Baseline).

Food group	Item	Unit	Males’ basic basket	Females’ basic basket
Grains and cereals (Minimally processed)	Porridge oats, unfortified	g	50	25
	Pasta, white, dried, raw	g	70	75
	Rice, white, long grain, raw	g	-	75
Grains and cereals (Processed)	Bread, wholemeal, and white	g	1029	574
	Breakfast cereal and muesli	g	100	90
Tubers (minimally processed and processed)	Potatoes (raw and ready to bake)	g	1740	725
Milk and other dairy	Milk	mL	2272	2000
	Cheese	g	160	120
	Sour-cream	g	125	-
	Yogurt (plain and flavored)	g	250	700
Eggs	Eggs	g	50	150
Meat and fish (minimally processed)	Red meat	g	400	210
	White meat	g	580	185
	Fish	g	460	200
Meat (processed)	Processed meats (ham, bacon, sausages)	g	298	149
Meat alternatives	Meat alternative (Quorn)	g	125	140
Legumes (minimally processed and processed)	Legumes (peas, baked beans)	g	610	560
Vegetables (minimally processed and processed)	Vegetables (spring onions, garlic, onions, tomatoes (fresh and canned), pepper, cucumbers, mushrooms, carrots, leafy vegetables)	g	2065	2782
Fruits (minimally processed)	Fruits (apples, bananas, pears, citrus fruit)	g	2160	1658
Fruits (processed)	Orange juice	mL	330	370
	Jam	g	20	20
Dried fruit and nuts	Dried fruit	g	90	-
	Nuts	g	-	50
Condiments, spices, and sauces	Salt and pepper	g	17	20
	Spices and condiments	g	32	146
	Sauces	mL	30	145
Added sugar	Honey	g	20	-
	Sugar, white	g	-	5
Butter, oils, and spreads	Butter and vegetable spreads	g	255	75
	Vegetable oil	mL	30	40
Snacks	Sweet snacks (biscuits, flapjack, cake, cereal bar)	g	366	143
	Savory snacks (oatcakes, crisps)	g	188	25
Ready meals	Ready meals (pizza, pies, canned soups)	g	1007	944
Coffee and tea	Coffee and tea	g	130	121

To investigate the impacts of substituting animal-sourced foods with NPBFs, we aimed to develop realistic dietary single-targeted substitution scenarios that reflect likely choices that an individual would make when intending to reduce either their processed meat and/or dairy consumption, while maintaining or improving the nutritional quality of their diet. We recognize that dietary transitions are often gradual, and account for the tendency of individuals to replace animal-sourced foods incrementally, typically 1 item at a time, rather than multiple changes at once [49,50]. This approach has been shown to increase the likelihood of sustaining dietary changes over time [50]. Accordingly, our scenarios involved a limited number of single-targeted substitutions, focusing on either 1 or a small selection of food items to mirror real-world behavior more accurately. Nine new baskets were created based on the original “basic basket” (baseline). All food items remained the same, and only single-targeted substitutions were chosen for each new basket: either replacing all dairy milk, all yogurt, or all processed meats in each basket, with their respective NPBF (see Table 2 for detailed quantities per basket). For each animal food product replaced, 3 substitution scenarios were considered from the most purchased NPBFs in the UK: *1*) the most popular NPBFs in their category (plant-based drinks, plant-based yogurt, or plant-based meat, respectively); *2*) the most inexpensive NPBF; and *3*) the most nutritionally balanced NPBFs. To select the most nutritionally balanced option, we used the UK nutrient profiling model (NPM) [51,52] as a proxy for healthiness. For consistency and comparability, we maintained the same weekly portion sizes as established in the “basic basket” (baseline) when constructing the “new basket.” For example, 110 g/wk of meat sausages were replaced by 110 g/wk of plant-based sausages (See Supplementary 2: Table S2.3 for detailed nutritional composition, environmental outcomes, and cost on a 100 g basis). We excluded plant-based cheese alternatives as a replacement for dairy cheese, due to their relatively poor nutritional quality among NPBF categories. Unlike other categories of NPBFs—which often contain vegetables and nuts, and dietary fiber—most plant-based cheese typically contains minimal whole food ingredients, and often lacks fiber entirely [28]. Therefore, we decided that currently swapping dairy cheese with plant-based cheese would not meaningfully support a healthier diet.

**TABLE 2:** New food baskets derived from baseline basket through single-item substitution: dairy milk, yogurt, or processed meats replaced with 1 novel plant- and fungi-based foods.

	Product in food basket	Unit	Males	Females
Baseline basket	Skimmed milk	mL	2272	2000
New basket 1	Almond-based drink (most popular), industry brand	g	2272	2000
New basket 2	Soy-based drink (most inexpensive), industry brand	g	2272	2000
New basket 3	Soy-based drink (nutritionally balanced), own-label	g	2272	2000
Baseline basket	Flavored yogurt	g	250	575
New basket 4	Soy-based yogurt (most popular), flavored, industry brand	g	250	575
New basket 5	Soy-based yogurt (most inexpensive), flavored, Industry brand	g	250	575
New basket 6	Soy-based yogurt (healthiest), flavored, own-label	g	250	575
Baseline basket	Natural yogurt	g	-	125
New basket 4	Soy-based yogurt (most popular), natural, industry brand	g	-	125
New basket 5	Soy-based yogurt (most inexpensive), natural, own-label	g	-	125
New basket 6	Soy-based yogurt (healthiest), natural, own-label	g	-	125
Baseline basket	Bacon	g	120	60
New baskets 7, 8, 9	Mycoprotein-based bacon (most popular), industry brand	g	120	60
Baseline basket	Ham	g	68	17
New basket 7	Mycoprotein-based ham (most popular), industry brand	g	68	17
New baskets 8 and 9	Mycoprotein-based ham (most inexpensive and nutritionally balanced), industry brand	g	68	17
Baseline basket	Sausages, pork	g	110	55
New basket 7	Soy-based sausages (most popular), industry brand	g	110	55
New basket 8	Mycoprotein-based sausages (most inexpensive), industry brand	g	110	55
New basket 9	Soy-based sausages (nutritionally balanced), Industry brand	g	110	55
Baseline basket	Chicken slices	g	-	17
New basket 7	Mycoprotein-based ham (most popular), industry brand	g	-	17
New baskets 8 and 9	Mycoprotein-based ham (most inexpensive and nutritionally balanced), industry brand	g	-	17

### Identification of the most purchased NPBF

To enable a realistic substitution within the “basic basket,” we identified the most frequently purchased (∼20‒25) plant-based meat, drink, and yogurt alternatives using Kantar’s Worldpanel Take Home data over a period of 52 wk from January 2019. This dataset offers a comprehensive overview of consumers’ purchasing habits at the household level in Great Britain (England, Wales and Scotland). We manually classified all NPBFs by category (e.g., plant-based meats, drinks, yogurts), with plant-based drinks and yogurts further divided into flavored and plain sub-products.

### Nutritional data

To standardize nutrient data from the “basic basket,” we used McCance and Widdowson’s composition of foods integrated dataset, which provides comprehensive nutrient profiles of foods in the UK food supply [53]. Using this dataset, we calculated total weekly nutrient intakes by multiplying the nutrient values for individual foods on a 100 g basis of food (See supplementary 2: Table S2.1 and S2.2) by their respective weekly quantities. These values were then aggregated across all items in the new baskets to estimate the weekly nutritional intake. To estimate the UK weekly recommended allowance for each nutrient of interest in each of the baskets, we multiplied the UK recommended daily allowances (RDAs) for adults by 7 [54]. We then used these values to compare the “basic basket” (baseline) to the new baskets, expressed as a proportion of the recommended weekly allowance. We also calculated the weekly average intake of red and processed meats, and fruit and vegetables, with the average daily intake of red and processed meats and fruit and vegetables for adults using the UK government dietary recommendations [55]) (See supplementary 1: Table S1.2).

To obtain up-to-date nutritional data for NPBFs, we conducted a cross-sectional supermarket survey of the most frequently purchased plant-based products identified in Kantar’s Worldpanel Take Home dataset. Nutrient data were collected from product packaging using the Open Food Facts application, a publicly accessible and crowdsourced nutrition database. The back-of-pack nutrient labels were scanned by 4 researchers (SNE, JTdS, AV, and EME) between May 2023 and December 2023. A pilot was conducted in March 2023 to help identify the supermarkets with the highest availability of relevant NPBF products.

### Nutritional quality indicators for NPBFs

In line with the UK Department of Health technical guidance [51,52,56], we used the UK NPM to evaluate the nutritional composition of the most purchased NPBFs identified in Kantar’s Worldpanel Take Home dataset. The UK NPM classifies foods or beverages as “healthy” or “less healthy,” based on their food nutritional composition per 100 g. It uses a points-based system that evaluates nutrients in 2 groups: “A” nutrients (energy, saturated fat, total sugar, and sodium) and “C” nutrients (fruit, vegetable, and nut content, fiber, and protein). The final NPM score is calculated by subtracting the total score for “C” from the total score for “A” nutrients. Foods scoring 4 or more points, or drinks scoring 1 or more points, are considered “less healthy.”

To convert the nutritional content of plant-based drinks from 100 mL to 100 g, we retrieved and used their specific gravity (density). The UK’s food composition table only includes specific gravity for soy drinks. Values for the other types of drinks were retrieved from the Australian food composition table (Supplementary 1: Table S1.1). To estimate free sugar content, we followed the methodology outlined in the 2018 NPM review: Annex A [51].

We also extracted 2 additional indicators for NPBFs from the Open Food Facts database: the Nutri-Score and NOVA classification category for each food. The Nutri-Score, developed under the leadership of the French Ministry of Health, is a front-of-pack labeling system rating products from “A” (highest nutritional quality score) to “E” (lowest nutritional quality score) within their respective categories [57]. We used the Nutri-Score to compare scores against the UK NPM results. NOVA classifies foods into 4 groups based on ingredients (e.g., food additives) and the level of processing. Ultraprocessed foods, which are often regarded as “unhealthy” or associated with poorer health outcomes, fall under category 4 [58].

### Environmental data

We estimated the environmental impacts (greenhouse gas emissions, water use, and land use) associated with the production of our foods in our analysis, using aggregated data from UK supermarkets shared via personal communications by Clark et al. [59]. Previously, Clark et al. [59] used these data to estimate the environmental impacts of different UK food products, including NPBFs.

To estimate the environmental impacts per 100 g or 100 mL of each product, we calculated weighted median values based on all products grouped within a specific supermarket “shelf” category (e.g., “oats & porridge,” “Brown & Wholemeal Bread,” “Milk”). In cases where the individual shelf labels were ambiguous (e.g., “Breakfast,” “Natural,” “Multipacks,” “Organic,” “View all”), we used the median values of the associated supermarket “aisle” category (e.g., “Yogurts,” “Milk, Butter & Eggs”). For example, the weighted median footprint for milk was derived from 423 different types of milk products from 2 shelf-categories: “Milk” and “Long life milk.” This process was applied consistently for all the other categories in the food basket.

### Price data

To estimate the cost of all products in the food baskets, we collected prices (without discounts or Clubcard prices) from 1 retailer (Tesco) on 13 March, 2024. For the most frequently purchased NPBFs identified in Kantar’s Worldpanel Take Home dataset, if price data was not available in Tesco, we collected prices from other supermarkets (Sainsbury’s, Asda, Morrisons, Waitrose, M&S) in March 2024.

### Data cleaning and analyses

**FIGURE 1:**
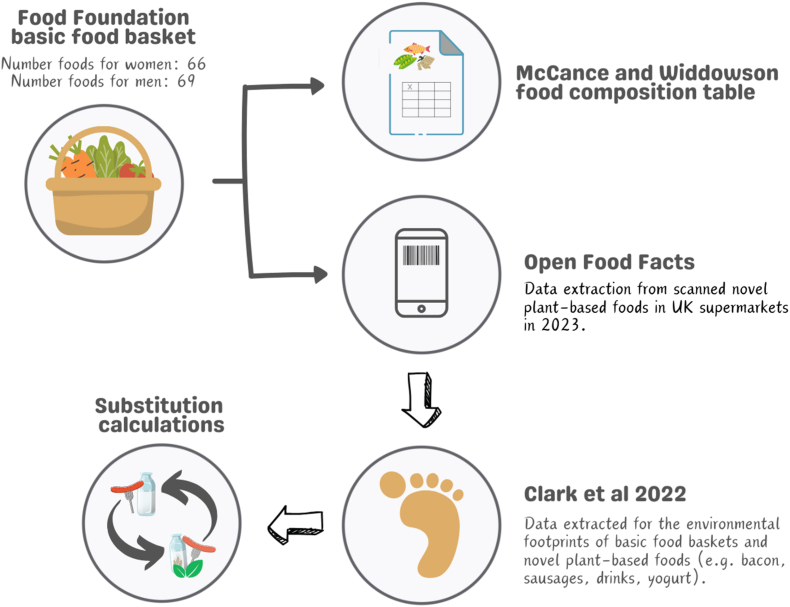
Simplified overview of the data linking process. Source: Authors with Canva.

Data cleaning and product category matching were conducted using RStudio v4.4.1 [60]. Figure 1 summarizes the process used to integrate all datasets. Nutrient, environmental, and price data for each food item were combined into a single table in Microsoft Excel to then calculate the weekly totals for each basket. We compared the “basic basket” (baseline) with the 3 NPBF substitution scenarios (most popular, most inexpensive, and nutritionally balanced) across processed meats, milk, and yogurt categories. For each basket, we calculated the percentage difference in nutrient content (including key macro- and micronutrients of relevance when substituting animal-based foods), environmental footprint (carbon emissions, water use, and land use), and total weekly cost relative to the baseline. We examined the direction and magnitude of changes for each outcome measure to identify which substitution strategy offered the greatest improvements in nutrition, sustainability, or affordability on a weekly basis. We also assessed whether nutrient concentrations met recommended dietary reference values by dividing weekly intake on a daily basis. All analyses were stratified by gender to reflect different nutritional requirements.

## Results

A total of 72 NPBFs were identified in the 2019 Kantar’s Worldpanel Take Home dataset (Supplementary 2: Table S2.6, S2.7 & S2.8). These comprised 25 frequently purchased types of plant-based meats, 25 types of plant-based drinks (all unflavored), and 22 types of plant-based yogurts (flavored and unflavored). All plant-based meats and drinks, and 82% of the plant-based yogurts, were classified as “healthy” when applying the NPM model. Wider differences were observed on the Nutri-Score: 88% of the plant-based meats and 40% of the plant-based drinks were classified as “A” products, with the remainder classified as “B” products. For plant-based yogurts, 50% were classified as “A,” 36% as “B,” and 14% as “C” products. On average across the 3 types of NPBFs, >88% fell under NOVA category 4 (ultraprocessed), 10% under NOVA category 3 (processed), and 3% under NOVA category 1 (minimally processed) (Supplementary 1: Figure S1.5, Figure S1.6, Figure S1.7).

**FIGURE 2:**
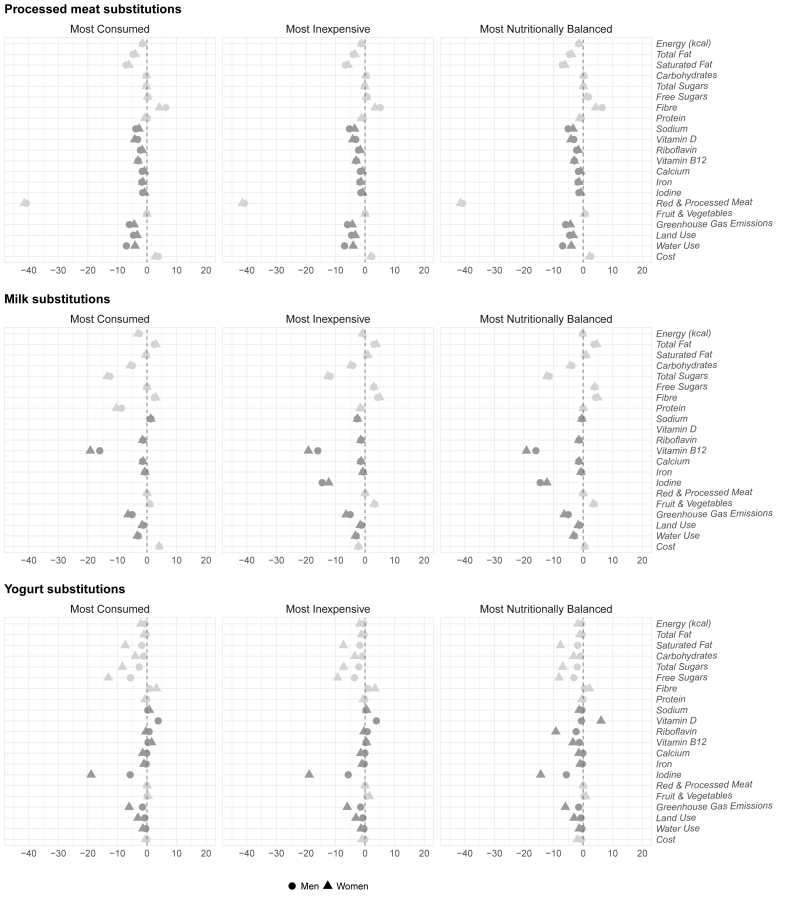
Percentage difference calculated from substituting processed meat, milk, and yogurt in a minimally acceptable, realistic, and affordable “basic basket” (baseline) with the most inexpensive, nutritionally balanced, and most popular novel plant-based alternatives to meat and dairy. Males’ and females’ basket are represented with circles and triangles, respectively. The grayscale color scheme is used solely to enhance the visibility of the different dietary and environmental markers, as well as cost. Panels (from top to bottom) are divided by meat, milk, and yogurt substitutions. Source: Authors.

Our analysis revealed a range of trade-offs and benefits associated with substituting processed meat, milk, and yogurt with like-for-like NPBFs. Figure 2 illustrates the percentage difference in each dietary and environmental indicator, as well as costs, when comparing the “basic basket” (baseline) with each of the new baskets (most inexpensive, most nutritionally balanced, and most popular) stratified by gender. Aggregated weekly values by food group for the nutrient content, environmental footprints, and total cost for both the baseline basket and new baskets are shown in Table 3 (males) and Table 4 (females). Detailed weekly values for all outcomes are shown in Supplementary 2: Table S2.4 (males) and Table S2.4 (females).

**TABLE 3:** Selected nutrients, environmental impact, and cost between the “basic basket” (baseline) compared with new baskets substituting processed meat, milk, and yogurt with the most popular, most inexpensive and healthy novel plant-based foods for males.

	Energy (kcal)	Saturated fat (g)	Total sugars (g)	Fiber (g)	Protein (g)	Sodium (mg)	Vitamin B12 (mcg)	Iron (mg)	Iodine (mcg)	GHG emissions (kg CO_2_ equivalent)	Land use (m^2^)	Water use (L)	Cost (GBP)
Grains and cereals (minimally processed and processed)	3242.92	17.73	41.00	75.59	119.52	4191.50	0.00	33.55	14.20	1.12	3.14	287.38	3.47
Tubers (minimally processed and processed)	2070.00	7.22	12.96	41.58	40.80	1090.60	0.00	7.64	17.00	1.42	3.50	101.47	2.20
Milk	772.48	2.95	109.06	0.00	79.52	999.68	18.18	0.68	681.60	4.54	2.20	374.97	2.41
*PB drink (most popular)*	*295.36*	*2.27*	*0.00*	*6.82*	*9.09*	*1272.32*	*8.63*	*0.00*	*0.00*	*2.13*	*1.29*	*208.23*	*4.77*
*PB drink (*most inexpensive*)*	*636.16*	*4.54*	*6.82*	*11.36*	*68.16*	*454.40*	*8.63*	*0.00*	*508.93*	*2.13*	*1.29*	*208.23*	*1.14*
*PB drink (nutritionally balanced)*	*749.76*	*4.54*	*9.09*	*11.36*	*79.52*	*908.80*	*8.63*	*0.00*	*508.93*	*2.13*	*1.29*	*208.23*	*2.73*
Other dairy: cheese and cream	1115.60	34.69	2.66	0.00	44.27	1569.30	3.84	0.98	48.00	1.03	1.09	87.71	2.41
Yogurt, flavored	272.50	5.03	41.50	NA	10.00	145.00	0.75	0.30	67.50	0.83	0.84	77.47	1.09
*PB yogurt (most popular), flavored*	*165.00*	*1.00*	*18.75*	*2.25*	*9.25*	*200.00*	*0.95*	*0.00*	*0.00*	*0.12*	*0.30*	*55.76*	*1.05*
*PB yogurt (*most inexpensive*), flavored*	*187.50*	*1.00*	*23.25*	*2.50*	*9.25*	*210.00*	*0.95*	*0.00*	*0.00*	*0.12*	*0.30*	*55.76*	*1.05*
*PB Yogurt (nutritionally balanced), flavored*	*187.50*	*0.75*	*24.25*	*1.25*	*9.50*	*60.00*	*0.00*	*0.00*	*0.00*	*0.12*	*0.30*	*55.76*	*0.80*
Eggs	65.50	1.26	0.00	0.00	6.30	77.00	1.35	0.86	25.00	0.16	0.17	14.42	0.25
Meat and fish (minimally processed)	2397.80	38.03	0.00	0.60	303.58	1338.80	29.44	15.95	133.60	24.87	41.37	2286.30	16.09
Meat (processed): bacon	258.00	7.44	0.00	0.00	19.80	1368.00	0.00	0.48	6.00	1.40	1.85	186.39	0.90
*PB bacon (most popular)*	*153.60*	*0.60*	*0.36*	*7.20*	*16.80*	*576.00*	*0.00*	*0.00*	*0.00*	*0.07*	*0.32*	*4.41*	*2.12*
Meat (processed): ham	72.76	0.75	0.68	0.07	12.51	544.00	0.68	0.48	3.40	0.64	0.87	87.47	1.09
*PB ham (most popular)*	*68.68*	*0.41*	*0.20*	*5.78*	*9.18*	*462.40*	*0.00*	*0.00*	*0.00*	*0.05*	*0.13*	*13.38*	*1.77*
*PB ham (most inexpensive and nutritionally balanced)*	*83.64*	*0.82*	*0.54*	*3.67*	*11.56*	*233.92*	*0.00*	*0.00*	*0.00*	*0.05*	*0.13*	*13.38*	*0.99*
Meat (processed): Sausages	339.90	10.07	3.08	3.08	13.09	517.00	1.10	0.99	7.70	0.97	1.27	142.14	0.67
*PB sausages (most popular)*	*168.30*	*0.99*	*0.77*	*7.26*	*20.46*	*616.00*	*0.00*	*0.00*	*0.00*	*0.05*	*0.18*	*12.38*	*0.94*
*PB sausages (*most inexpensive*)*	*217.80*	*1.54*	*1.65*	*6.05*	*12.10*	*528.00*	*0.00*	*0.00*	*0.00*	*0.05*	*0.18*	*12.38*	*0.81*
*PB sausages (nutritionally balanced)*	*167.20*	*0.55*	*4.07*	*9.46*	*12.32*	*572.00*	*0.00*	*0.00*	*0.00*	*0.05*	*0.18*	*12.38*	*1.02*
Meat alternatives	91.25	0.50	0.50	10.38	17.50	375.00	0.38	0.75	NA	0.13	0.49	10.18	2.68
Vegetables (minimally processed and processed)	446.82	0.80	81.18	38.31	18.16	175.08	0.00	7.12	39.52	2.52	2.04	409.09	5.51
Legumes (minimally processed and processed)	502.30	2.48	19.03	31.53	38.29	526.10	0.00	14.32	8.20	0.66	4.10	166.92	1.34
Fruits (minimally processed)	1174.15	1.42	270.71	32.92	15.68	16.60	0.00	2.99	55.60	1.25	1.62	557.25	4.51
Fruits (processed)	169.40	0.00	42.10	0.00	2.85	10.70	0.00	0.27	4.70	0.20	0.27	58.34	0.50
Dried fruit and nuts	247.50	NA	62.46	NA	2.43	17.10	0.00	1.98	NA	0.24	0.69	163.64	0.17
Condiments, spices, and sauces	272.64	3.40	2.64	7.48	7.90	1115.10	1.54	3.97	7.00	0.10	0.15	24.59	1.09
Butter, oil, and spreads	1316.40	43.38	3.00	0.00	0.27	1015.80	0.14	0.05	17.10	0.63	1.26	58.78	1.34
Added sugar	57.60	0.00	15.28	0.00	0.08	2.20	0.00	0.08	0.00	0.05	0.05	21.00	0.18
Snacks	2373.83	37.86	134.72	20.78	34.20	2346.24	0.00	14.06	9.99	1.41	2.17	250.09	5.95
Ready meals	926.14	15.10	29.24	6.10	25.48	2426.42	2.35	4.30	42.46	1.46	1.86	183.98	2.37
Total weekly “basic basket” (baseline)^a^	18,207.99	**230.08**	**871.80**	**268.41**	**816.70**	**19,891.52**	**59.74**	**113.17**	**1188.57**	**47.74**	**73.73**	**5560.98**	**57.05**
New basket 1: most popular PB drink	17,730.87	229.40	762.74	275.23	746.27	20,164.16	50.20	112.49	506.97	45.34	72.82	5394.24	59.41
New basket 2: most inexpensive PB drink	18,071.67	231.67	769.56	279.77	805.34	19,346.24	50.20	112.49	1015.90	45.34	72.82	5394.24	55.77
New basket 3: *nutritionally balanced* PB drink	18,185.27	231.67	771.83	279.77	816.70	19,800.64	50.20	112.49	1015.90	45.34	72.82	5394.24	57.36
New basket 4: most popular PB yogurt	18,100.49	226.06	849.05	270.66	815.95	19,946.52	59.94	112.87	1121.07	47.04	73.19	5539.27	57.01
New basket 5: most inexpensive PB yogurt	18,122.99	226.06	853.55	270.91	815.95	19,956.52	59.94	112.87	1121.07	47.04	73.19	5539.27	57.01
New basket 6: *nutritionally balanced* PB yogurt	18,122.99	225.81	854.55	269.66	816.20	19,806.52	58.99	112.87	1121.07	47.04	73.19	5539.27	56.76
New basket 7: most popular PB meat	17,927.91	213.83	869.37	285.50	817.74	19,116.92	57.96	111.23	1171.47	44.91	70.37	5175.15	59.22
New basket 8: most inexpensive PB meat	17,992.37	214.79	870.59	282.18	811.76	18,800.44	57.96	111.23	1171.47	44.91	70.37	5175.15	58.32
New basket 9: *nutritionally balanced* PB meat	17,941.77	213.80	873.01	285.59	811.98	18,844.44	57.96	111.23	1171.47	44.91	70.37	5175.15	58.53

Abbreviations: CO_2_, carbon dioxide; GHG, greenhouse gas; PB, plant based; GBP, Great British Pound; NA, not applicable.

^a^ baseline basket total

**TABLE 4:** Selected nutrients, environmental impact, and cost between the “basic basket” (baseline) compared with new baskets substituting processed meat, milk, and yogurt with the most popular, most inexpensive, and healthy novel plant-based foods for females.

	Energy (kcal)	Saturated fat (g)	Total sugars (g)	Fiber (g)	Protein (g)	Sodium (mg)	Vitamin B12 (mcg)	Iron (mg)	Iodine (mcg)	GHG emissions (kg CO_2_ equivalent)	Land use (m^2^)	Water Use (L)	Cost (GBP)
Grains and cereals (minimally processed and processed)	2236.85	5.27	19.00	49.11	79.91	2524.58	0.00	21.72	6.60	0.96	2.14	355.64	1.88
Tubers (minimally processed and processed)	792.50	2.31	5.70	16.65	16.25	329.65	0.00	2.95	7.25	0.60	1.46	41.49	0.81
Milk	680.00	2.60	96.00	0.00	70.00	880.00	16.00	0.60	600.00	3.99	1.93	330.08	2.12
*New basket 1: almond-based drink (most popular), industry brand*	*260.00*	*2.00*	*0.00*	*6.00*	*8.00*	*1120.00*	*7.60*	*0.00*	*0.00*	*1.88*	*1.13*	*183.30*	*4.20*
*New basket 2: soy-based drink (*most inexpensive*), industry brand*	*560.00*	*4.00*	*6.00*	*10.00*	*60.00*	*400.00*	*7.60*	*0.00*	*448.00*	*1.88*	*1.13*	*183.30*	*1.00*
*New basket 3: soy-based drink (nutritionally balanced), own-label*	*660.00*	*4.00*	*8.00*	*10.00*	*70.00*	*800.00*	*7.60*	*0.00*	*448.00*	*1.88*	*1.13*	*183.30*	*2.40*
Other dairy: cheese and cream	499.20	26.02	0.12	0.00	30.48	867.60	2.88	0.36	36.00	0.45	0.46	37.29	1.34
Yogurt, natural	98.75	2.39	9.75	0.00	7.13	100.00	0.25	0.13	78.75	0.42	0.42	38.73	0.71
*New basket 4: soy-based yogurt (most popular), natural, industry brand*	*63.75*	*0.50*	*2.63*	*1.25*	*5.00*	*125.00*	*0.48*	*0.00*	*0.00*	*0.06*	*0.15*	*27.88*	*0.53*
*New basket 5: soy-based yogurt (*most inexpensive*), natural, own-label*	*51.25*	*0.50*	*0.00*	*1.13*	*5.38*	*50.00*	*0.00*	*0.00*	*0.00*	*0.06*	*0.15*	*27.88*	*0.28*
*New basket 6: soy-based yogurt (nutritionally balanced), natural, own-label*	*52.50*	*0.50*	*0.00*	*1.38*	*5.88*	*40.00*	*0.48*	*0.00*	*56.25*	*0.06*	*0.15*	*27.88*	*0.36*
Yogurt, flavored	626.75	11.56	95.45	NA	23.00	333.50	1.73	0.69	155.25	1.91	1.92	178.17	2.50
*New basket 4: soy-based yogurt (most popular), flavored, industry brand*	*379.50*	*2.30*	*43.13*	*5.18*	*21.28*	*460.00*	*2.19*	*0.00*	*0.00*	*0.28*	*0.69*	*128.24*	*2.42*
*New basket 5: soy-based yogurt (*most inexpensive*), flavored, industry brand*	*431.25*	*2.30*	*53.48*	*5.75*	*21.28*	*483.00*	*2.19*	*0.00*	*0.00*	*0.28*	*0.69*	*128.24*	*2.42*
*New basket 6: soy-based yogurt (nutritionally balanced), flavored, own-label*	*431.25*	*1.73*	*55.78*	*2.88*	*21.85*	*138.00*	*0.00*	*0.00*	*0.00*	*0.28*	*0.69*	*128.24*	*1.84*
Eggs	196.50	3.78	0.00	0.00	18.90	231.00	4.05	2.58	75.00	0.47	0.50	43.25	0.75
Meat and fish (minimally processed)	1019.90	19.25	0.00	0.24	127.03	596.45	12.20	5.84	54.00	11.91	20.69	1124.02	6.77
Meat (processed): bacon	175.80	4.98	0.00	0.00	14.04	834.00	0.60	0.42	4.20	0.70	0.92	93.19	0.29
*PB bacon (most popular)*	*76.80*	*0.30*	*0.18*	*3.60*	*8.40*	*288.00*	*0.00*	*0.00*	*0.00*	*0.03*	*0.16*	*2.21*	*1.06*
Meat (processed): ham	18.19	0.19	0.17	0.02	3.13	136.00	0.17	0.12	0.85	0.16	0.22	21.87	0.27
*PB ham (most popular)*	*17.17*	*0.10*	*0.05*	*1.45*	*2.30*	*115.60*	*0.00*	*0.00*	*0.00*	*0.01*	*0.03*	*3.34*	*0.44*
*PB ham (*most inexpensive *and nutritionally balanced)*	*20.91*	*0.20*	*0.14*	*0.92*	*2.89*	*58.48*	*0.00*	*0.00*	*0.00*	*0.01*	*0.03*	*3.34*	*0.25*
Meat (processed): sausages	169.95	5.03	1.54	1.54	6.55	258.50	0.55	0.50	3.85	0.48	0.64	71.07	0.34
*PB sausages (most popular)*	*84.15*	*0.50*	*0.39*	*3.63*	*10.23*	*308.00*	*0.00*	*0.00*	*0.00*	*0.02*	*0.09*	*6.19*	*0.47*
*PB sausages (*most inexpensive*)*	*108.90*	*0.77*	*0.83*	*3.03*	*6.05*	*264.00*	*0.00*	*0.00*	*0.00*	*0.02*	*0.09*	*6.19*	*0.41*
*PB sausages (nutritionally balanced)*	*83.60*	*0.28*	*2.04*	*4.73*	*6.16*	*286.00*	*0.00*	*0.00*	*0.00*	*0.02*	*0.09*	*6.19*	*0.51*
Meat (processed): slices	19.38	0.07	0.03	0.05	3.94	98.60	0.00	0.07	0.85	0.16	0.16	8.51	0.10
*PB ham (most popular)*	*17.17*	*0.10*	*0.05*	*1.45*	*2.30*	*115.60*	*0.00*	*0.00*	*0.00*	*0.01*	*0.03*	*3.34*	*0.44*
*PB ham (*most inexpensive *and nutritionally balanced)*	*20.91*	*0.20*	*0.14*	*0.92*	*2.89*	*58.48*	*0.00*	*0.00*	*0.00*	*0.01*	*0.03*	*3.34*	*0.25*
Meat alternatives	102.20	0.56	0.56	11.62	19.60	420.00	0.42	0.84	NA	0.15	0.54	11.40	3.00
Vegetables (minimally processed and processed)	589.67	1.53	87.72	47.02	34.46	258.01	0.00	13.11	56.37	3.65	3.53	725.48	11.80
Legumes (minimally processed and processed)	456.80	1.26	22.88	28.08	31.04	1045.60	0.00	10.16	3.20	0.65	3.82	88.40	0.66
Fruits (minimally processed)	1040.93	0.98	236.89	24.49	14.48	8.78	0.00	2.97	45.62	1.14	1.38	704.09	2.85
Fruits (processed)	183.00	0.00	45.50	0.00	3.09	11.90	0.00	0.30	5.10	0.21	0.30	62.87	0.58
Dried fruit and nuts	290.50	3.86	2.80	NA	11.90	2.50	0.00	1.47	8.00	0.13	0.38	90.91	1.00
Condiments, spices, and sauces	1121.42	10.94	26.31	4.20	8.20	5804.65	0.61	2.72	28.05	0.29	0.54	68.79	2.32
Butter, oil, and spreads	613.85	9.05	0.98	0.00	0.00	361.50	0.00	0.05	0.00	0.30	0.95	29.39	0.44
Added sugar	19.70	0.00	5.25	0.00	0.00	0.25	0.00	0.01	0.00	0.01	0.01	3.68	0.01
Snacks	766.06	11.75	33.96	5.91	10.43	858.27	0.04	3.07	2.22	0.37	0.70	72.89	2.12
Ready meals	1627.78	29.11	25.29	12.79	61.65	2740.96	4.38	7.52	67.85	2.01	2.82	261.74	6.51
Coffee and tea	30.00	0.00	0.00	0.00	5.92	32.40	0.00	1.84	0.00	1.84	2.32	14.12	0.86
**Total weekly “basic basket” (baseline)**	**13,375.68**	**152.48**	**715.90**	**201.71**	**601.12**	**18,734.70**	**43.87**	**80.01**	**1239.01**	**32.98**	**48.75**	**4477.08**	**50.02**
New basket 1: most popular PB drink	12,955.68	151.88	619.90	207.71	539.12	18,974.70	35.47	79.41	639.01	30.87	47.95	4330.31	52.10
New basket 2: most inexpensive PB drink	13,255.68	153.88	625.90	211.71	591.12	18,254.70	35.47	79.41	1087.01	30.87	47.95	4330.31	48.90
New basket 3: *nutritionally balanced* PB drink	13,355.68	153.88	627.90	211.71	601.12	18,654.70	35.47	79.41	1087.01	30.87	47.95	4330.31	50.30
New basket 4: most popular PB yogurt	13,093.43	141.33	656.45	208.13	597.27	18,886.20	44.55	79.20	1005.01	30.99	47.25	4416.29	49.75
New basket 5: most inexpensive PB yogurt	13,132.68	141.33	664.18	208.58	597.64	18,834.20	44.08	79.20	1005.01	30.99	47.25	4416.29	49.50
New basket 6: *nutritionally balanced* PB yogurt	13,133.93	140.76	666.48	205.96	598.72	18,479.20	42.37	79.20	1061.26	30.99	47.25	4416.29	49.02
New basket 7: most popular PB meat	13,187.65	143.21	714.82	210.22	596.68	18,234.80	42.55	78.91	1229.26	31.56	47.13	4297.52	51.44
New basket 8: most inexpensive PB meat	13,219.88	143.69	715.43	208.56	593.69	18,076.56	42.55	78.91	1229.26	31.56	47.13	4297.52	50.99
New basket 9: *nutritionally balanced* PB meat	13,194.58	143.19	716.64	210.26	593.80	18,098.56	42.55	78.91	1229.26	31.56	47.13	4297.52	51.10

Abbreviations: CO_2_, carbon dioxide; GHG, greenhouse gas; PB, plant based; GBP; NA, not applicable.

Overall, the impact of NPBF substitution on dietary quality and recommended weekly allowance was largely positive, with only marginal negative nutritional effects for some substitutions (marked in dark red in Table 5). For example, single-targeted substitutions of processed meats with NPBF alternatives yielded notable benefits for both males and females, including increased fiber intake and reduced energy intake. There were modest increases in fruit and vegetables intake across most new baskets for both genders, regardless of the product substituted. Across all new baskets, we observed consistent reductions in energy density compared to the baseline baskets for both males and females. Changes in total fat, total sugar, free sugars, and protein varied depending on the specific NPBF used as a substitution. Most new baskets showed reductions in saturated fat, except in cases when milk was replaced with the most inexpensive or most nutritionally balanced alternative.

**TABLE 5:**
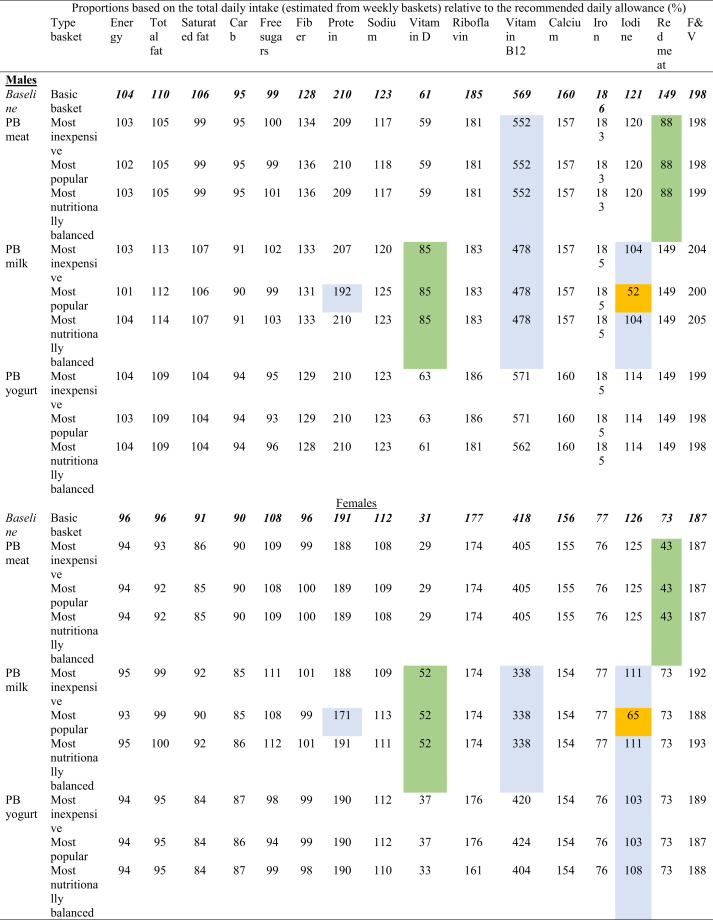
**Comparison of the daily proportions of the United Kingdom government dietary recommendations for males and females between the “basic basket” (baseline) and new baskets**. Each new basket substituted processed meat, milk, and yogurt with most inexpensive, most nutritionally balanced, and the most popular novel PB alternatives to meat and dairy. Positive outcomes in comparison to baseline basket larger than 15% are highlighted in green, whereas negative outcomes are highlighted in light blue. Notable reductions below the recommended weekly allowance are highlighted in orange, indicating the potential risk to contribute to micronutrient deficiencies.

Abbreviations: F&V, fruit and vegetables; PB, plant based; BB, basic basket; I, Most inexpensive; P, Most popular; NB, Most nutritionally balanced

^1^Red meat includes processed meat.

Although not all NPBFs were fortified, and some like-for-like NPBFs had lower micronutrient content compared to their animal-sourced counterparts, their inclusion had minimal impact on overall micronutrient intake across the full dietary baskets. This was especially true when replacing processed meats with NPBFs which are typically lower in that usually contain less micronutrients and are higher in fat [61]. When NPBFs with more favorable nutritional profiles were selected—especially when replacing meat and yogurt with the most nutritionally balanced NPBFs—diets continued to meet all micronutrient weekly RDAs targets. However, a notable reduction in iodine was observed when replacing milk with the most affordable plant-based alternative.

Environmental outcomes showed consistent improvements across all substitution scenarios. For males, dietary greenhouse gas emissions decreased by ‒1.47% to ‒5.95% for land use by ‒0.72% to ‒4.55% and water use by ‒0.39% to ‒6.94%. For females, these figures were ‒4.31% to ‒6.41%, ‒1.64% to ‒3.32%, and ‒1.36% to ‒4.01%, respectively.

Regarding the cost, the most popular plant-based drinks and meats tended to increase overall dietary expenses. In contrast, yogurt substitutions showed modest economic savings across the 3 new baskets: ‒£0.04 to ‒£0.29 per week for males (‒0.06% and ‒0.50%) and ‒£0.27 to ‒£1.01 per week for females (‒0.54% and ‒2.01%). Conversely, all processed meat substitutions led to increased weekly costs: £1.27‒£2.17 per week for males (+2.23% and +3.80% increase) and £0.97‒£1.42 per week for females (+1.94% and +2.84%).

## Discussion

In recent years, both the supply and demand of NPBFs have increased rapidly in the UK. Given NPBFs’ increasing availability, it is crucial to evaluate the implications of NPBFs on nutritional adequacy, environmental sustainability, and overall dietary costs. This study adopted a practical single-targeted substitution approach, exploring the replacement of specific animal-sourced foods in a realistic, minimally acceptable, nutritionally adequate, and affordable “basic basket” (baseline) with the most popular, inexpensive, and nutritionally balanced NPBFs.

The findings showed that single-targeted substitutions of animal-based products with NPBFs within a balanced diet can deliver meaningful environmental benefits without compromising overall nutritional adequacy—except in the case of the most popular plant-based drink, which contributed lower concentrations of iodine and protein compared to the baseline basket. Although the costs of substituting animal-sourced foods with NPBFs remain relatively high, particularly for plant-based meat products, which increased the weekly basket cost by £0.97‒£2.17, some plant-based drinks (the most inexpensive and nutritionally balanced alternatives) and all plant-based yogurt alternatives demonstrated promising cost advantages over their animal-sourced counterparts. However, ongoing cost disparities, especially for plant-based meats, may pose barriers to adoption, particularly between individuals from lower socioeconomic groups, and may discourage broader consumer uptake despite nutritional and environmental benefits.

Without policy efforts to improve the affordability of NPBFs, particularly in the context of meat and dairy reduction strategies, there is a risk of missing a critical opportunity to advance both net zero and public health objectives.

### Research in context

A substantial body of evidence demonstrates that shifts toward plant-based diets, either incorporating more plant-based whole foods (e.g., whole grains, legumes) or substituting animal-based foods with plant-based whole foods, are healthy and sustainable [13,[62], [63], [64], [65]]. However, these studies typically do not include NPBFs in their analyses. Our study uniquely examines multiple, single-targeted NPBF substitutions within a minimally acceptable, realistic, and affordable “basic basket” as a baseline. Unlike previous product-level comparisons [[28], [29], [30], [31], [32], [33], [34]] or complete replacement models [66], we simultaneously assess nutrition composition, environmental impacts, and cost using real product data for the UK’s most consumed NPBFs. We focus on targeted replacements (e.g., processed meat) rather than aggregated categories, providing practical evidence for achievable dietary transitions.

Previous research has noted that many NPBFs are lower in key micronutrients typically found in animal-sourced foods, such as vitamin B12, iodine, iron, and calcium [31]. Our study supports these concerns, particularly for plant-based meat alternatives that are unfortified and contribute to less micronutrient intakes. However, despite these lower contents, the overall dietary quality remained largely adequate, with most nutrients’ recommended weekly allowances met or exceeded across baskets. These findings highlight the importance of evaluating dietary substitutions in context—accounting not only for product-level characteristics but also for broader dietary patterns, accessibility, and environmental outcomes, especially as consumers of NPBF also consume animal-sourced foods in their diets [42,43].

Contrary to common concerns, this study found minimal nutritional impacts when substituting selected animal-source foods with NPBFs in a minimally acceptable “food basket.” Although this basket does not align with national dietary guidelines, these findings offer a proxy for understanding the potential benefits if individuals made small changes in their current diets. Given that only 0.01% of the UK population currently adheres to the national food-based dietary guidelines [17] and persistent sociodemographic inequalities in dietary adherence remain [48], the potential nutritional and environmental benefits of the evaluated substitutions in real-world diets could be even more substantial since the average dietary quality of the UK population tends to be less healthy than our current “basic basket.” This is particularly relevant in light of a recent report suggesting that even modest reductions in meat consumption, if widely adopted, could contribute substantially to improved population health, reduced environmental impacts, and potential cost savings for the National Health Service [67].

We also contribute to the ongoing debate about the classification of NPBFs as ultraprocessed. Currently, most NPBFs fall under NOVA category 4 due to their ingredient lists (e.g., presence of food additives) and processing techniques. Although ultraprocessed food consumption has been widely associated with adverse health outcomes [68,69], evidence suggests that NPBFs may not carry the same risks when considered separately [70,71]. In our analysis, most NPBFs did not exhibit the typical nutrient profiles of ultraprocessed foods, such as being energy-dense, low in fiber, and high in sugar, saturated fat, and sodium—features typically associated with unhealthy ultraprocessed foods. On the contrary, some products even increased the intake of beneficial dietary components such as fruits, vegetables, and hence fiber.

Most new baskets with single-targeted dairy substitutions resulted in cheaper food baskets, with the notable exception of processed meat alternatives. This is particularly concerning given that individuals from lower socioeconomic backgrounds, who predominantly consume processed meats [7,44], face the greatest cost barriers to healthier and more sustainable dietary transitions. However, there are encouraging signs of change. A 2022 Dutch survey found that price parity between meat and plant-based meat alternatives was narrowing, with some NPBFs such as plant-based cheese and soy drinks already cheaper than their animal-based counterparts [72]. By 2024, all NPBFs in the Netherlands had become cheaper than meat and dairy, resulting in an estimated 20% reduction in the average Dutch shopping basket—if all animal-sourced foods were to be replaced by NPBFs [73]. Although price parity is observed in some countries like the Netherlands and Germany, 2 recently published articles on the cost of plant-based meats and drinks have shown that, on average, these products continue to be more expensive than their animal-based equivalents in the UK and other European countries [33,74]. Although projections suggest that price parity between NPBFs and animal-sourced foods will be achieved by 2035 [75], the current cost-of-living crisis poses a notable barrier. Many households may be unable, or unwilling, to pay a premium for NPBFs, limiting their potential to support dietary shifts that contribute to environmental sustainability.

### Relevance for policy and practice

Single-targeted substitutions with the most purchased NPBFs in the UK can provide substantial environmental advantages without undermining nutritional sufficiency. However, our findings on the cost of NPBFs indicate the need for regulations to make NPBFs more affordable to support dietary change behavior, particularly for those in the lowest income brackets. Current evidence shows that regular consumers of NPBFs are more likely to be females, younger, have higher education, and come from affluent households [76]. Price parity may facilitate NPBF’s broader consumer uptake, without excluding those socioeconomically disadvantaged.

Micronutrient deficiencies remain a common concern when transitioning to more plant-forward diets. However, the extent of these concerns depends on the degree to which animal-sourced foods are reduced. Like in our study, modeled flexitarian dietary patterns in the United States, for example, have not been associated with negative dietary quality [63], suggesting that partial substitutions may mitigate these risks. For individuals following more restrictive dietary patterns, such as vegans, where micronutrient deficiency risks are higher, targeted interventions could ensure adequate micronutrient intake. For example, in 2024, the WHO/Europe called for action to ensure appropriate iodine fortification in plant-based drinks and other products [77]. Similar fortification strategies, whether through voluntary industry efforts or mandatory national food standards, could be extended to other micronutrients across different types of NPBFs.

Finally, given the limited evidence on the bioavailability and bioaccessibility of micronutrients in NPBFs and the relationship around the level of processing of NPBFs, clinical trials are needed to fully comprehend the role of processing for satiating effects, changes in the food matrix, micronutrient absorption, protein quality, and by-products formed during packaging and processing. This would support a more nuanced classification system that distinguishes between different types of ultraprocessed foods, particularly the “healthiest” and “least healthy” NPBFs, to guide consumers, public procurement, and inform policy and practice on plant-based transitions. This is crucial given that 53% of the British population, according to a survey of 2136 participants, support a tax on companies producing ultraprocessed foods [78]. However, imposing a blanket tax on ultraprocessed foods could inadvertently hinder the potential positive outcomes found in this study, thereby challenging progress toward more plant-forward diets.

### Strengths and limitations

Our study has several strengths. First, this study introduces a novel approach by assessing nutritional and environmental indicators alongside the price of NPBFs that UK consumers may choose, identifying the most inexpensive, nutritionally balanced, and popular options from a real take home purchase behavior panel across the UK. In terms of behavior change interventions, these findings offer realistic and long-term strategies to reduce environmental footprints without compromising consumers’ sensory experience and the nutritional quality of their diets. Although findings show that the price and micronutrient content of NPBFs still present obstacles to adopting these products, NPBFs offer nutritional and environmental benefits. Public bodies could therefore focus on improving affordability and establishing food standards for NPBFs to encourage industry-wide fortification. The baseline baskets were standardized with national nutritional data from the UK food composition table, whereas the NPBFs data were collected in 2023, as very few products are included in the UK food composition table. Lastly, the environmental data shared by Clark et al. [59] utilized in this study is the best available footprint metrics for supermarket products, making the study’s outcomes the most realistic to date.

Our study also has several limitations. We used a minimally acceptable and affordable food basket as our baseline to substitute single-targeted animal-sourced foods with NPBFs that were commonly consumed in 2019 by the UK population. Because estimating people’s regular purchasing behaviors is complicated, particularly for the lower income bracket in the UK, this option offers a reasonable option of what a socially acceptable, realistic, and affordable diet looks like and how each of the substitutions impacts nutritional and environmental outcomes and cost. However, it is important to note 2 main limitations: first, the basket does not provide the same nutritional composition recommended in the national food-based dietary guidelines, but instead it provides a more realistic and socially acceptable diet; second, we only examined a few of the many possible substitutions that consumers may have. Therefore, the chosen “basic basket” is a reasonable approximation of realistic dietary behaviors for the study population, even though it may not be representative across different sociodemographic groups in current UK diets. Future modeling studies should assess common dietary patterns across different sociodemographic groups with different types of substitutions to have a better estimation of the trade-offs and possibilities.

Price data for the basket was collected from a single supermarket without any deals or discounts. Therefore, prices for animal-sourced foods and NPBFs may vary in other scenarios. For comparison purposes for the “new basket,” we conducted the analysis using the same weekly portion size (e.g., 80 g sausages for 80 g of plant-based sausages) established in the “basic basket” to substitute each animal-sourced food. Therefore, this work could be revised by using the recommended portion size for each NPBF required to sustain a healthy diet.

For the iron daily requirements for females, we used the highest value (14.8 mg/d) for females of reproductive age; therefore, the baseline and new baskets were all below the RDA. If we had used the lower bound value (8.7 mg/d), all baskets would have been above the weekly RDAs, as was the case for males. However, further research is needed to understand the bioavailability of iron in NPBFs. Vitamin D was also lower than the weekly RDAs on the baseline basket; this may be because the baseline basket also accounted for vitamin D absorption from the sun. Furthermore, weekly RDAs were estimated for the average population, so results may be different for individuals with different dietary requirements, such as children, pregnant females, seniors, and individuals with underlying conditions. The use of NOVA categorization is highly debated in the scientific community due to the accumulative evidence of the negative health effects of ultraprocessed foods [69,[79], [80], [81]] and the use of subjective terms (e.g., “wholesome,” “natural,” “mass-produced”) to classify foods in the 4 categories [26,82]. A more granular classification system is essential for better understanding the health implications of the level of processing in NPBFs.

Fully considering all environmental impacts in food systems and diets is already complex. Therefore, we focused on 3 key environmental impact categories: greenhouse gas emissions, land use, and water use. Although dietary greenhouse gas emissions are more commonly evaluated, prioritizing these 3 categories not only ensured better comparability with prior research on NPBFs but also made it more feasible to interpret multiple environmental, nutritional, health, and cost outcomes together. However, new research should consider other environmental categories to capture trade-offs that may have been overlooked. Furthermore, our dietary footprints for NPBFs were estimated from the median values for each NPBF category (e.g., plant-based meats, drinks, yogurts). However, it was not possible to disaggregate them by type of product [e.g., plant-based bacon, drink, sausage, yogurt (plain)] and by primary ingredient (e.g., soy, pea, mycoprotein). This could limit the accuracy of the environmental footprint estimates for NPBFs. Furthermore, Clark’s environmental data does not incorporate postproduction processing, packaging, and transportation. This omission may lead to a substantial underestimation of the environmental impacts, and therefore, more accurate data are required. Because environmental footprints are subject to differences in methodologies, especially for water use and land use, we observed a large difference between some of the assessed NPBFs in our study (See Supplementary 1: Table S1.3) in comparison to others (See Supplementary 1: Table S1.4 for weighted medians for NPBF categories segregated by primary ingredient using UK data collected from a recently published systematic review [28]).

## Conclusion

This study demonstrated that single-targeted substitutions of either all processed meats, milk, or yogurt with commonly purchased NPBFs lead to meaningful environmental gains, including greenhouse gas emission, water use, and land use reductions, without compromising the overall nutritional adequacy of diets. In several cases, particularly with the most nutritionally balanced plant-based drinks and yogurts, substitutions even enhanced key micronutrient intake such as iodine. However, the nutritional composition of NPBFs varies widely, and our findings highlight the need for careful selection and possible fortification of NPBFs to avoid unintended nutritional shortfalls.

In terms of cost, plant-based drinks and yogurts offer cheaper alternatives to their animal-sourced counterparts; however, plant-based meat alternatives remain more expensive. To fully harness the health and environmental benefits of NPBFs, urgent efforts are needed to improve their affordability and make them more accessible.

These findings underscore the potential of NPBFs to support healthier and more sustainable diets, provided that nutritional quality and cost barriers are addressed through coordinated policy, product innovation, and consumer guidance.

## Author contributions

The authors’ responsibilities were as follows – SNE, PS: designed the research and methodology; TC: supported with the statistical methodology and visualization code; SNE, AGZ-O: analyzed data; SNE: conducted the research, wrote the article, and had primary responsibility for the final content; SNE, JTdS, AV, EME: collected nutrient and/or price data; AR: supported in the visualization of supplementary figures; and all authors: read and approved the final manuscript.

## Data availability

Aggregated data described in the manuscript will be made available upon request, pending application and approval.

## Declaration of AI and AI-Assisted Technologies in the Writing Process

During the preparation of this work, the author(s) used ChatGPT in order to assist with improving the language and readability of the manuscript. After using this tool/service, the author(s) reviewed and edited the content as needed and take(s) full responsibility for the content of the publication.

## Funding

This work was supported by a research grant from the National Institute for Health Research, Health Protection Research Unit PhD Studentship in Environmental Change and Health (grant NIHR200909), and the Af Jochnick Foundation. The funders had no role in the conception, design, execution, or approval of this work.

## Conflict of interest

The authors report no conflicts of interest.
