# Supplementary material for: Novel Plant and fungi-based Alternatives Support Nutritional Adequacy of Diets and Reduce Their Environmental Impacts
Source: Curr Dev Nutr. 2026 Mar 12;10(4):107669. doi: 10.1016/j.cdnut.2026.107669 (PMC13091752; doi:10.1016/j.cdnut.2026.107669)
Supplement: multimedia component 1 [file mmc1.docx]

**Supplementary Materials 1 for:**

Novel plant-based alternatives support nutritional adequacy of diets and reduce their environmental impacts

Sarah Nájera Espinosa,*^1,2^ Arli G. Zarate-Ortiz,^1,2^ Genevieve Hadida,^1,2^ Jacqueline Tereza Da Silva,^3^ Alexander Vonderschmidt,^3^ Edith Monica Esievo,^1^ Tony Carr,^1,2^ Anouk Reuzé^1,2^ and Pauline Scheelbeek*^1,2^

^1^ Department of Population Health, London School of Hygiene and Tropical Medicine, London, United Kingdom

^2^ Centre on Climate Change and Planetary Health, London School of Hygiene and Tropical Medicine, London, United Kingdom

^3^ Division of Global Academy of Agriculture and Food Systems, University of Edinburgh

*Corresponding authors email: [sarah.najeraespinosa@lshtm.ac.uk](mailto:sarah.najeraespinosa@lshtm.ac.uk); [pauline.scheelbeek@lshtm.ac.uk](mailto:pauline.scheelbeek@lshtm.ac.uk)

# List of tables

[Table S1. 1: Specific gravity (density) for plant-based drinks. 2](#_Toc218084184)

[Table S1. 2: UK recommended daily and weekly allowance from the UK government dietary recommendations (3, 4). Weekly allowance was estimated by multiplying the recommended daily allowance by 7. 2](#_Toc218084185)

[Table S1. 3: Weighted median environmental footprints for plant-based alternatives to meat and dairy per 100 g/ml of food estimated from Clark et al. 2022 (5). 3](#_Toc218084186)

[Table S1. 4: Weighted median environmental footprints from UK articles for novel plant-based alternatives to meat and dairy per 100 g/ml of food estimated from Nájera Espinosa et al 2024 (6). 4](#_Toc218084187)

# List of figures

[Figure S1. 1: Environmental impacts of the substituted animal-sourced foods (e.g. processed meat, milk, and yogurt) with novel plant-based alternatives. 5](#_Toc218084191)

[Figure S1. 2: Nutrient profile and cost of plant-based drinks by main ingredient. 6](#_Toc218084192)

[Figure S1. 3: Nutrient profile and cost of plant-based drinks by main ingredient. 7](#_Toc218084193)

[Figure S1. 4: Nutrient profile and cost of plant-based meats by main ingredient. 8](#_Toc218084194)

[Figure S1. 5: Overview of the nutrient profiling model, nutri-score and NOVA category for plant-based drinks. 9](#_Toc218084195)

[Figure S1. 6: Overview of the nutrient profiling model, nutri-score and NOVA category for plant-based yogurts. 9](#_Toc218084196)

[Figure S1. 7: Overview of the nutrient profiling model, nutri-score and NOVA category for plant-based yogurts. 10](#_Toc218084197)

Table S1. 1: Specific gravity (density) for plant-based drinks.

| **Plant-based drink** | **Specific gravity (Density)** | **Source code** | **Source name** | **Source** |
| --- | --- | --- | --- | --- |
| **Almond and Hazelnut** | 1.05 | F009824 | Almond beverage, no added sugar, unfortified | Australian FCT(1) |
| **Blends (Cereals & coconut or nuts)** | 1.05 | F006132, F007632 | Average: Oat beverage and rice beverage | Australian FCT(1) |
| **Coconut** | 1.03 | F009812 | Coconut beverage, unfortified | Australian FCT(1) |
| **Oat** | 1.05 | F006132 | Oat beverage, fluid, unfortified | Australian FCT(1) |
| **Rice** | 1.05 | F007632 | Rice beverage, fluid, added calcium | Australian FCT(1) |
| **Soy** | 0.99 | 12-524 | Milk, soya, non-dairy alternative to milk, unsweetened, fortified | UK FCT (2) |
| Abbreviations: FCT, Food composition table | | | | |

Table S1. 2: UK recommended daily and weekly allowance from the UK government dietary recommendations (3, 4). Weekly allowance was estimated by multiplying the recommended daily allowance by 7.

|  | **UK Recommended daily allowance** | | **UK Recommended weekly allowance** | |
| --- | --- | --- | --- | --- |
|  | **Men** | **Women** | **Men** | **Women** |
| **Energy** | 2500.00 | 2000.00 | 17500.00 | 14000.00 |
| **Fat** | 97.00 | 78.00 | 679.00 | 546.00 |
| **Saturated fat** | 31.00 | 24.00 | 217.00 | 168.00 |
| **carbohydrate** | 333.00 | 267.00 | 2331.00 | 1869.00 |
| **Free sugars** | 33.00 | 27.00 | 231.00 | 189.00 |
| **Fibre** | 30.00 | 30.00 | 210.00 | 210.00 |
| **Protein** | 55.50 | 45.00 | 388.50 | 315.00 |
| **Sodium** | 2400.00 | 2400.00 | 16800.00 | 16800.00 |
| **Vitamin D** | 10.00 | 10.00 | 70.00 | 70.00 |
| **Riboflavin** | 1.30 | 1.10 | 9.10 | 7.70 |
| **Vitamin B12** | 1.50 | 1.50 | 10.50 | 10.50 |
| **Calcium** | 700.00 | 700.00 | 4900.00 | 4900.00 |
| **Iron** | 8.70 | 14.80 | 60.90 | 103.60 |
| **Iodine** | 140.00 | 140.00 | 980.00 | 980.00 |
| **Red and processed meats** | 70.00 | 70.00 | 490.00 | 490.00 |
| **Fruit and vegetables** | 400.00 | 400.00 | 2800.00 | 2800.00 |

Table S1. 3: Weighted median environmental footprints for plant-based alternatives to meat and dairy per 100 g/ml of food estimated from Clark et al. 2022 (5).

| **Category** | **Name** | **Number products** | **Greenhouse gas emissions**  **(Kg CO_2_ eq)** | **Land Use**  **(m^2^)** | **Water use (Liters)** |
| --- | --- | --- | --- | --- | --- |
| **Plant-based meat** | Breaded meat | 64 | 0.094 | 0.341 | 12.674 |
|  | Bacon | 9 | 0.057 | 0.269 | 3.677 |
|  | Burgers | 19 | 0.053 | 0.189 | 13.005 |
|  | Meatballs | 5 | 0.103 | 0.689 | 4.613 |
|  | Meat slices (Ham) | 19 | 0.080 | 0.190 | 19.675 |
|  | Mince, fillets & pieces | 152 | 0.107 | 0.325 | 16.099 |
|  | Sausages | 23 | 0.043 | 0.166 | 11.254 |
|  | Sausages & Burgers, combined | 187 | 0.103 | 0.336 | 9.051 |
|  | Unknown type, average general | 1120 | 0.098 | 0.260 | 13.892 |
| **Plant-based dairy** | Drinks | 624 | 0.094 | 0.057 | 9.165 |
|  | Yogurts | 197 | 0.049 | 0.121 | 22.302 |

Table S1. 4: Weighted median environmental footprints from UK articles for novel plant-based alternatives to meat and dairy per 100 g/ml of food estimated from Nájera Espinosa et al 2024 (6).

| **Category** | **Name** | **Food group** | **Greenhouse gas emissions**  **(# products)** | **Greenhouse gas emissions**  **(Kg CO_2_ eq)** | **Land use**  **(# products)** | **Land use**  **(m^2^)** | **Water use**  **(# products)** | **Water use (Liters)** |
| --- | --- | --- | --- | --- | --- | --- | --- | --- |
| **Plant-based meat** | Breaded meats | Mycoprotein | 2 | 0.17 | 1 | 0.00 | 1 | 6.90 |
|  | Burger | Fruits & Vegetables | 1 | 0.17 |  |  | 1 | 7.00 |
|  | Burger | Legumes | 2 | 0.48 |  |  | 2 | 540.00 |
|  | Ham | Mycoprotein | 1 | 0.26 | 1 | 0.00 | 1 | 6.50 |
|  | Mince, fillets & pieces | Cereals & Grains | 8 | 0.38 |  |  | 8 | 720.00 |
|  | Mince, fillets & pieces | Legumes | 3 | 0.30 | 1 | 0.00 | 3 | 270.00 |
|  | Mince, fillets & pieces | Mycoprotein | 8 | 0.12 | 8 | 0.08 | 8 | 5.45 |
|  | Sausages | Legumes | 1 | 0.11 |  |  | 1 | 11.00 |
|  | Sausages | Mycoprotein | 3 | 0.13 | 3 | 0.00 | 3 | 12.60 |
| **Plant-based dairy** | Cheese | Coconut | 8 | 0.27 | 8 | 0.18 | 8 | 2.95 |
|  | Drinks | Cereals & Grains | 2 | 0.04 |  |  | 1 | 32.00 |
|  | Drinks | Legumes | 5 | 0.05 |  |  | 5 | 74.00 |

Figure S1. 1: Environmental impacts of the substituted animal-sourced foods (e.g. processed meat, milk, and yogurt) with novel plant-based alternatives.

*Note: Environmental impacts of the substituted animal-sourced foods (e.g. processed meat, milk, and yogurt) in each new basket for their like-for-like affordable, healthy, and most popular novel plant-based alternatives. Each data point shows the median value, while error bars show the 25^th^ and 75^th^ interquartile ranges. Animal-sourced foods (baseline) are marked with an X and shown in red. Novel plant-based foods are illustrated in turquoise, with shapes indicating their category: circles for affordable, squares for healthy, triangles for most popular, and diamonds for those that are both affordable and healthy.*

Figure S1. 2: Nutrient profile and cost of plant-based drinks by main ingredient.


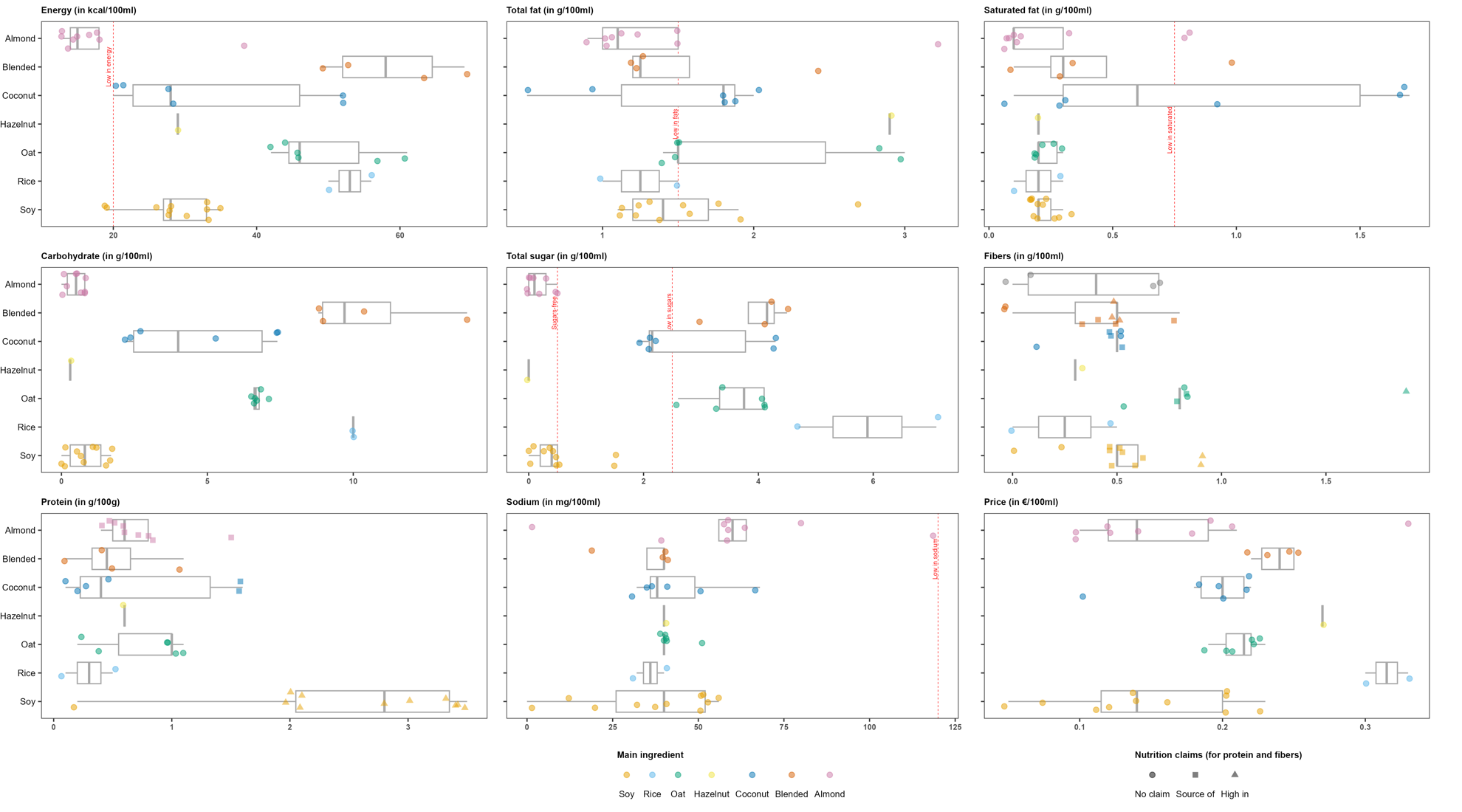


Figure S1. 3: Nutrient profile and cost of plant-based drinks by main ingredient.


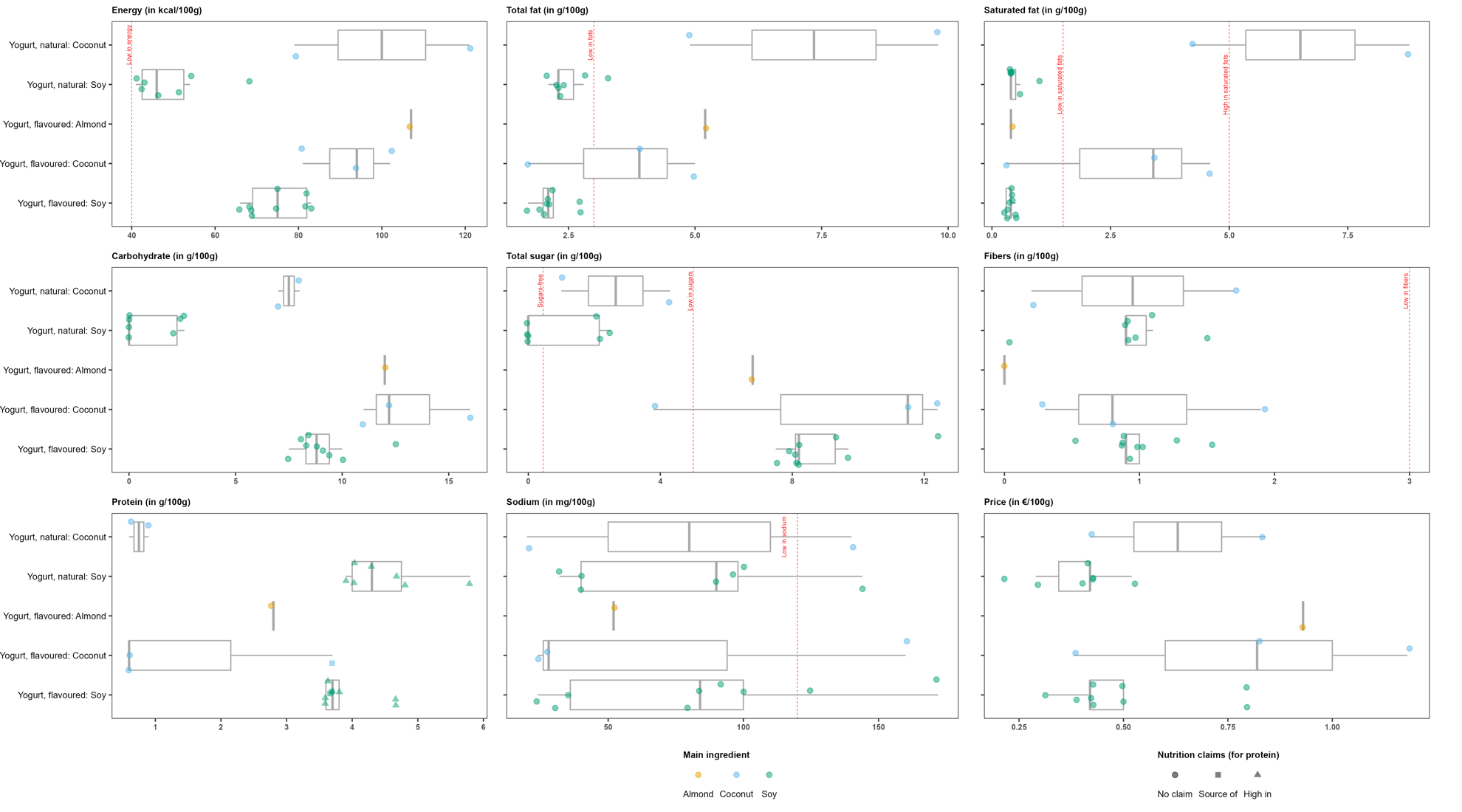


Figure S1. 4: Nutrient profile and cost of plant-based meats by main ingredient.


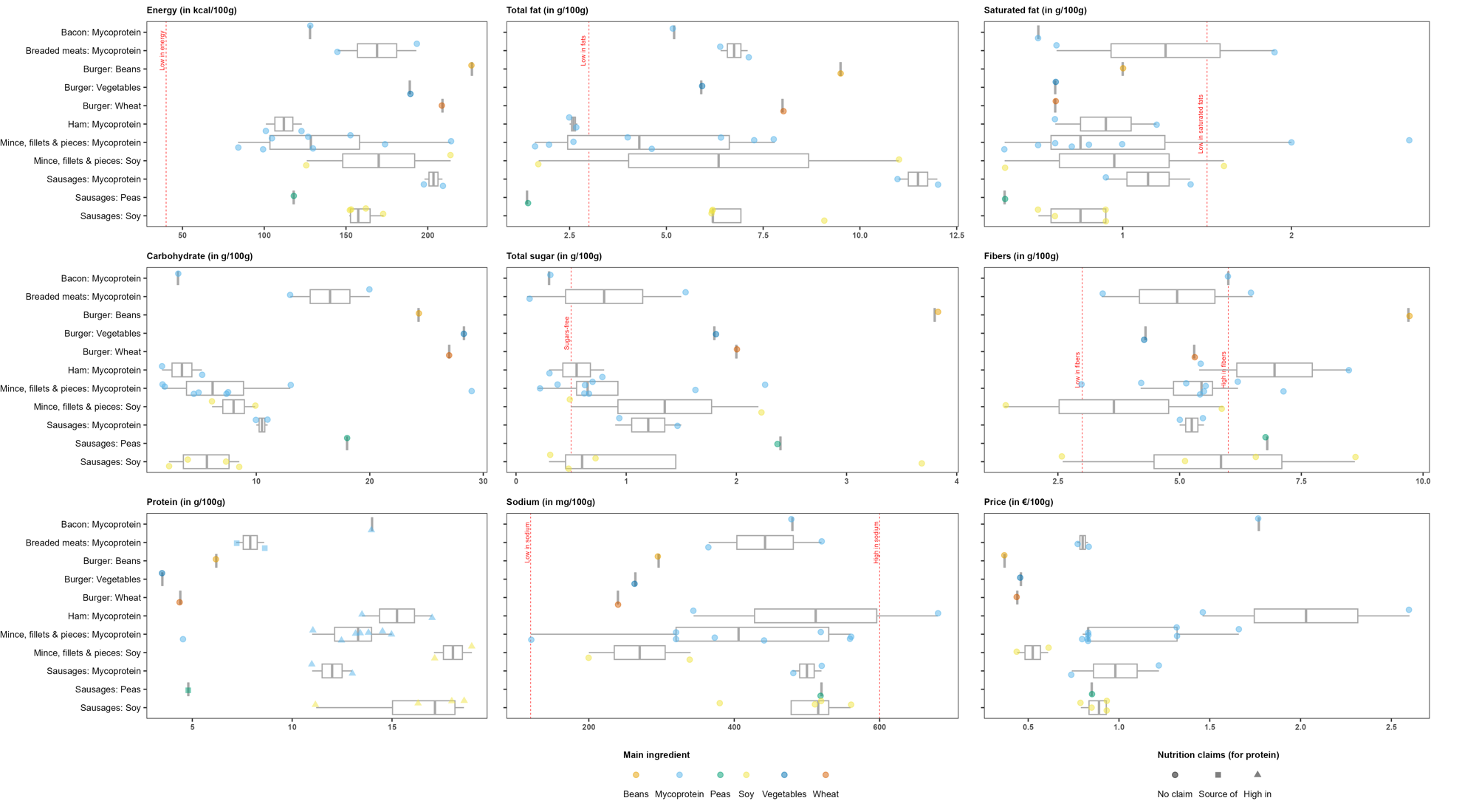


Figure S1. 5: Overview of the nutrient profiling model, nutri-score and NOVA category for plant-based drinks.


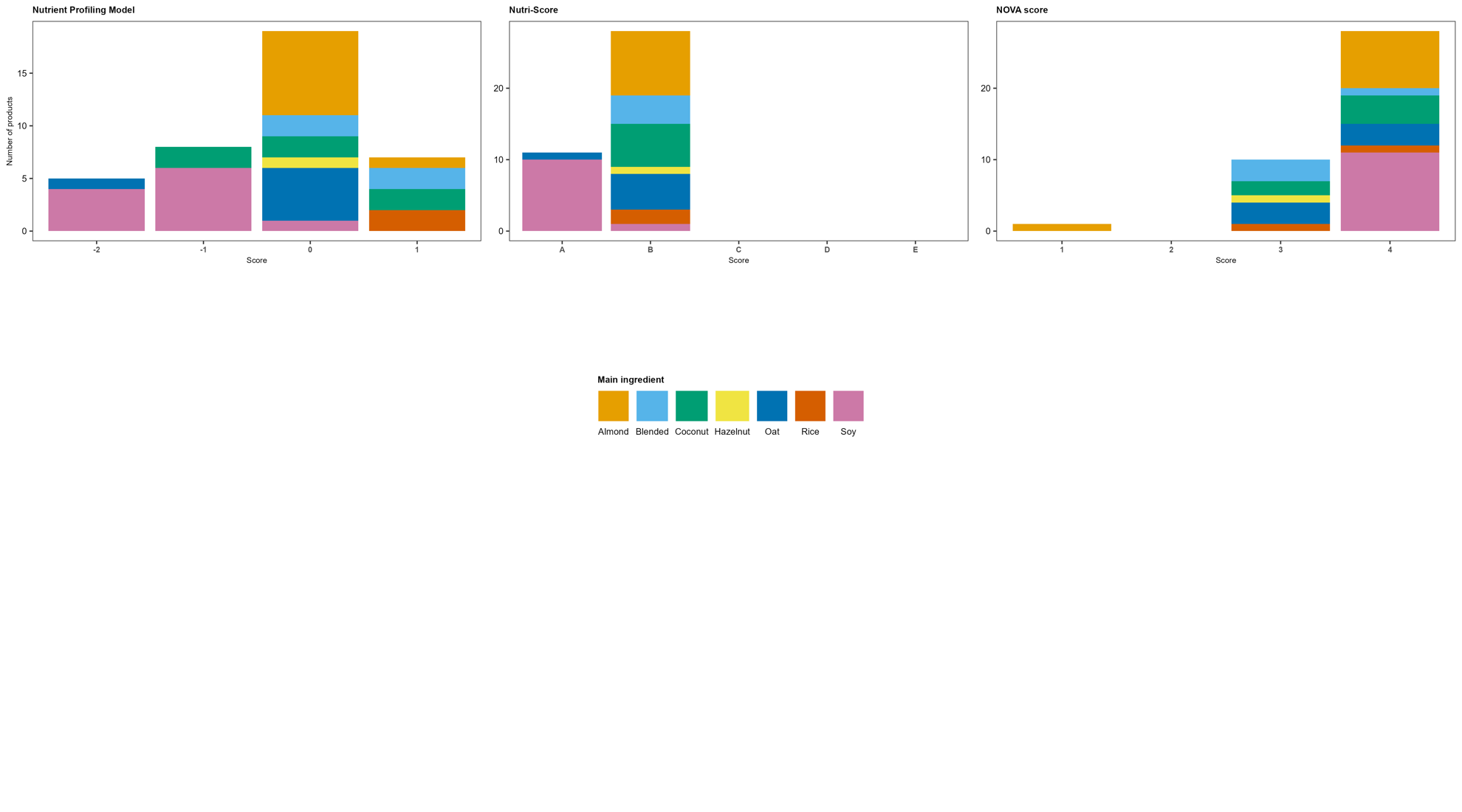


Figure S1. 6: Overview of the nutrient profiling model, nutri-score and NOVA category for plant-based yogurts.


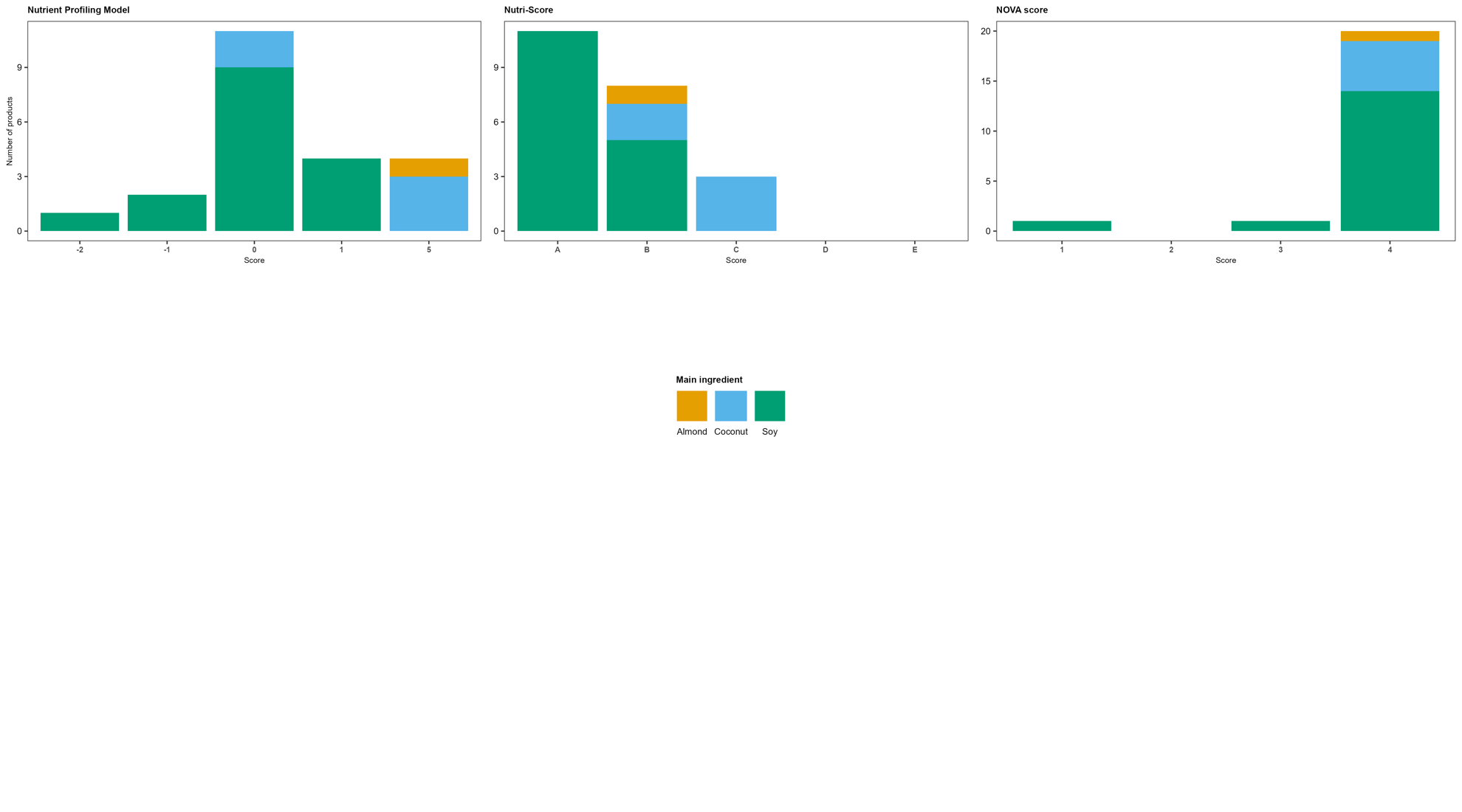


Figure S1. 7: Overview of the nutrient profiling model, nutri-score and NOVA category for plant-based yogurts.


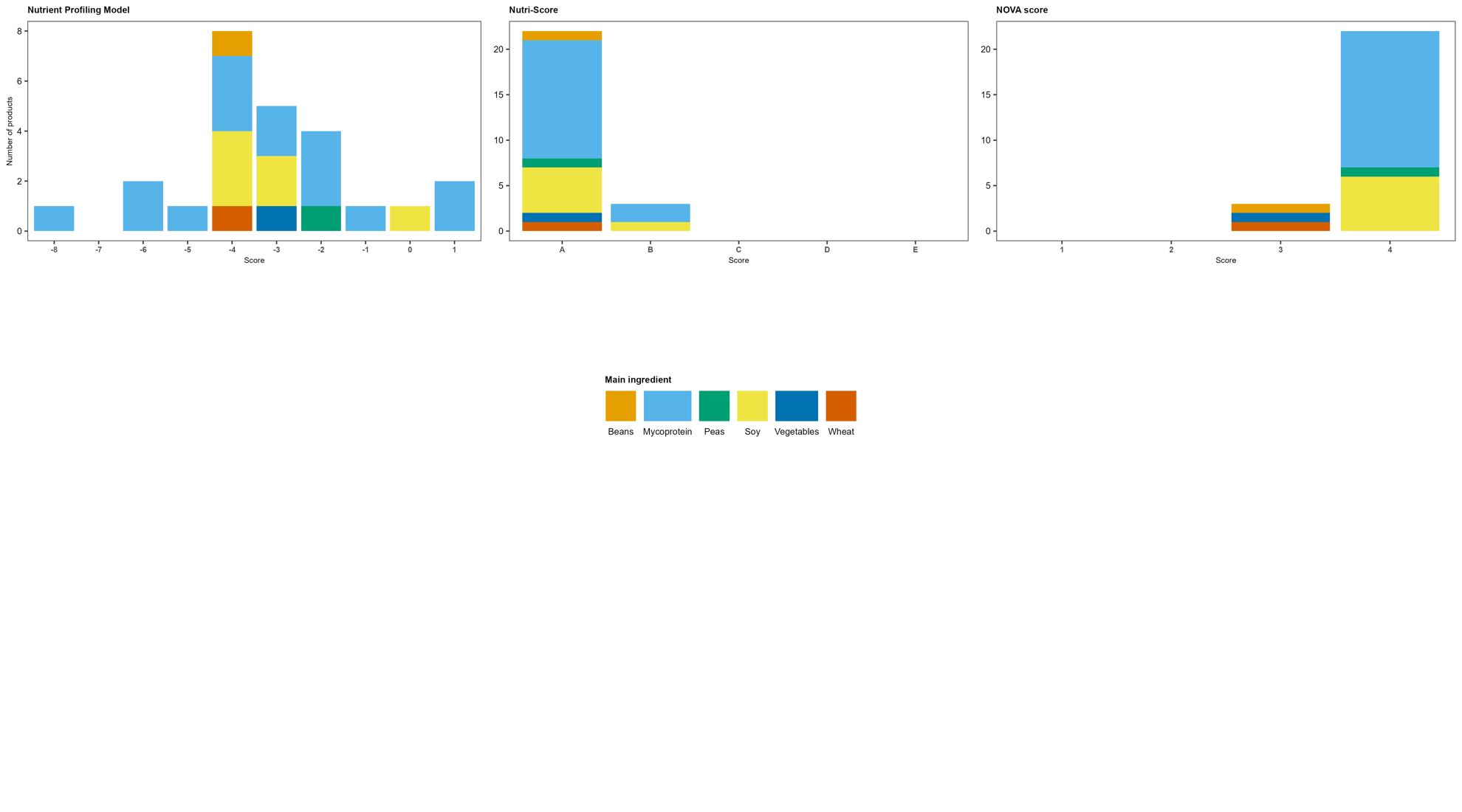


# References

1. FSANZ. Australian Food Composition Database - Release 2.0: Food Standards Australia and New Zealand; 2023 [cited 2024 July]. Available from: <https://www.foodstandards.gov.au/science-data/monitoringnutrients/afcd>.

2. Public Health England. McCance and Widdowson’s The Composition of Foods Integrated Dataset (CoFID) 2021 2021 [cited 2024 July]. Available from: <https://www.gov.uk/government/publications/composition-of-foods-integrated-dataset-cofid>.

3. Public Health England. Government Dietary Recommendations: Government recommendations for energy and nutrients for males and females aged 1 – 18 years and 19+ years. London: 2016.

4. Public Health England. The Eatwell Guide booklet. In: Disparities OfHIa, editor. United Kingdom2018.

5. Clark M, Springmann M, Rayner M, Scarborough P, Hill J, Tilman D, et al. Estimating the environmental impacts of 57,000 food products. Proc Natl Acad Sci U S A. 2022;119(33):e2120584119. Epub 20220808. doi: 10.1073/pnas.2120584119. PubMed PMID: 35939701.

6. Nájera Espinosa S, Hadida G, Jelmar Sietsma A, Alae-Carew C, Turner G, Green R, et al. Mapping the evidence of novel plant-based foods: A systematic review of nutritional, health, and environmental impacts in high-income countries. Nutrition Reviews. 2024;nuae031. doi: <https://doi.org/10.1093/nutrit/nuae031>.
